# Supplementary material for: Comprehensive Annotation and Functional Exploration of MicroRNAs in Lettuce
Source: Front Plant Sci. 2021 Dec 24;12:781836. doi: 10.3389/fpls.2021.781836 (PMC8739914; doi:10.3389/fpls.2021.781836)
Supplement: Supplementary file 3 [file Data_Sheet_9.ZIP › GSM2230751.plot/Lsa-miRN1652b_Lsat_1_v5_gn_1_77600.1_83_TPlot.pdf]

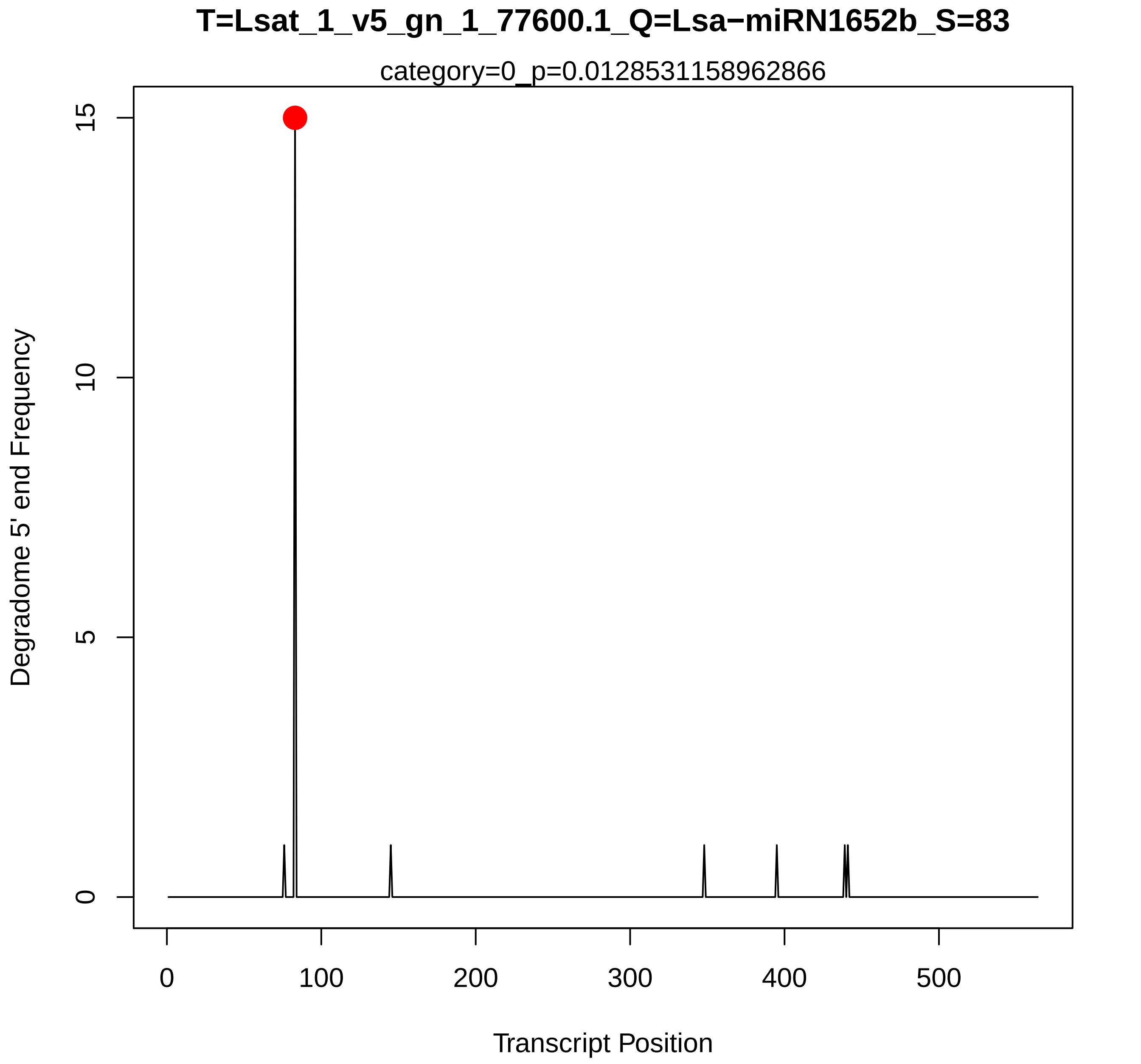

T=Lsat_1_v5_gn_1_77600.1_Q=Lsa−miRN1652b_S=83
category=0_p=0.0128531158962866
Degradome 5' end Frequency
0
5
10
15
0
100
200
300
400
500
Transcript Position
